# Harnessing artificial intelligence for engineering extracellular vesicles

**DOI:** 10.20517/evcna.2025.35

**Published:** 2025-09-02

**Authors:** Hui Lu, Jin Zhang, Tianzhuo Shen, Wenbing Jiang, Han Liu, Jiacan Su

**Affiliations:** ^1^Institute of Translational Medicine, Shanghai University, Shanghai 200444, China.; ^2^MedEng-X Institute, Shanghai University, Shanghai 200444, China.; ^3^National Center for Translational Medicine (Shanghai) SHU Branch, Shanghai University, Shanghai 200444, China.; ^4^Organoid Research Center, Shanghai University, Shanghai 200444, China.; ^5^College of Medicine, Shanghai University, Shanghai 200444, China.; ^6^Department of Cardiology, Wenzhou Hospital of Integrated Traditional Chinese and Western Medicine, Wenzhou 325000, Zhejiang, China.; ^7^Su Zhou Innoovation Center of Shang Hai University, Suzhou 215000, Jiangsu, China.; ^8^Sanming Second Hospital, Sanming 366000, Fujian, China.; ^9^Department of Orthopedics, Xinhua Hospital, Shanghai Jiao Tong University School of Medicine, Shanghai 200092, China.; ^#^Authors contributed equally.

**Keywords:** Extracellular vesicles, artificial intelligence, machine learning, drug delivery, targeted therapy

## Abstract

Extracellular vesicles (EVs) are a type of cell-released phospholipid bilayer nanoscale carrier. However, research on EVs encounters several challenges, such as their heterogeneity, the complexities associated with their isolation and identification, the necessity for engineering optimization, and the limitations in exploring their mechanisms. The advancement of artificial intelligence (AI) technologies offers new opportunities for EV research. Here, the definition and brief history of AI, as well as types and common models of machine learning, are first introduced, and the interactions between AI, machine learning, and deep learning are explored. The article then discusses in detail a variety of applications of AI in EV research, including the use of AI for target identification and selective delivery of EVs, the design and optimization of drug delivery systems, the mapping of cellular communication networks, the analysis of multi-omics data, and synthetic biology-based research on EVs. These applications demonstrate the potential of AI in advancing EV research and applications. Finally, we offer an outlook on the major challenges and future prospects of AI. Overall, the introduction of AI technologies has provided new perspectives and tools for the study of EVs, which is expected to enhance the application of EVs in disease diagnosis and treatment.

## INTRODUCTION

Extracellular vesicles (EVs) are nanoscale particles enclosed by membranes, secreted by cells^[1-3]^. EVs provide insight into the metabolic status and functional activities of the source cells in various pathological conditions^[4-6]^. Based on their biogenesis and size, EVs can be classified into three subgroups: microvesicles, apoptotic bodies, and exosomes derived from endosomal cells^[7,8]^. Microvesicles, ranging in size from 20 nanometers up to 1,000 nanometers, primarily comprise cell membrane markers. In contrast, the diameter of apoptotic bodies ranges from 500 nanometers to 5,000 nanometers^[9-11]^, which is the largest category of cell release involved in the elimination of apoptotic cells. On the other hand, exosomes, ranging in size from 30 nanometers to 200 nanometers, are released through the fusion of endosomal multivesicular compartments with the cell membrane, showing good therapeutic potential in drug delivery because they carry important signal molecules^[12-15]^. While EVs exhibit therapeutic promise, challenges such as limited material sources, inadequate targeting ability of natural EVs^[16,17]^, and unstable circulation must be addressed. It is imperative to develop more efficient methodologies for EV-based disease diagnosis and therapy. In recent years, engineered methods have been employed to enhance the targeting effectiveness of EVs^[18-22]^.

Artificial Intelligence (AI) is a field of computer science that builds systems to perform human cognitive tasks such as visual recognition, speech interpretation, decision making, and language understanding. To improve the predictive capabilities, information classification, and complex problem-solving skills of AI systems, the development process must be underpinned by extensive datasets. AI systems are developed with extensive datasets to facilitate their ability to make predictions, categorize information, and tackle intricate challenges. Machine learning, an advanced technology stemming from AI advancements, is an interdisciplinary domain^[21,23]^. In recent years, AI has advanced rapidly and become closely connected with EV research, where algorithms can process large datasets from experiments to enable efficient and detailed analysis^[24]^, and through this, AI can evaluate structural and functional features of EVs to provide insights into disease processes and support the development of new therapies. Its ability in analysis and classification helps build accurate EV-based drug delivery systems, and studies have also shown its strong predictive performance in protein folding, evolutionary biology, and multi-omics research^[23,25-29]^, forming a solid basis for the development of precision drug delivery strategies using EVs.

This review summarizes the main challenges in EV research and introduces AI with its definition, background, and key branches of machine learning. It describes the links between AI, machine learning, and deep learning and presents applications in EV studies that include target identification, controlled delivery, drug system design, mapping of cellular networks, multi-omics analysis, and synthetic biology. These examples highlight the value of AI in advancing EV research. The review also examines issues such as lack of standards, ethical concerns, data quality, privacy, and model bias, and outlines possible solutions. Continued progress in AI is expected to support EV research and accelerate biomedical innovation.

## KEY CHALLENGES OF EVS

EVs are nanoscale membrane-bound particles secreted by cells and widely distributed in bodily fluids, including blood, saliva, and cerebrospinal fluid^[30-32]^. They play crucial roles in both physiological and pathological processes, such as intercellular communication, immune regulation, and tissue repair, by transporting bioactive molecules, including proteins, nucleic acids, and lipids^[33-35]^. EVs have recently attracted major attention in biomedical research for their potential in diagnostics, regenerative medicine and drug delivery^[1,36]^. However, the considerable heterogeneity of EVs, the intricate processes underlying their biogenesis, and the technical constraints of large-scale isolation methods pose major obstacles to both in-depth investigation and clinical translation.

### Heterogeneity of EVs

The release of EVs from diverse cell populations reflects their intrinsic heterogeneity. Depending on the cellular origin, EVs differ substantially in both molecular profiles and biological roles. For example, vesicles secreted by tumor cells contain distinct protein and RNA signatures compared with those produced by normal cells^[7]^. The molecular composition of EVs differs among cell lineages, mirroring the physiological status of their cells of origin and shaping their roles in intercellular signaling. Even within a single cell type, EVs can vary in size and the abundance of proteins, nucleic acids, and lipids, which contributes to substantial functional diversity^[37]^. Their size ranges from about 30 nanometers to several micrometers, which affects their distribution and targeting *in vivo*. The amounts and types of proteins, RNAs, and lipids also depend on cell origin and condition, shaping signaling activity. This heterogeneity is a key factor in the functional complexity of EVs^[38]^. Under normal conditions, EVs support cell communication, immune regulation, and tissue repair, whereas in disease, they may alter their function; for example, tumor-derived EVs can promote cancer growth, spread, and immune escape^[39]^. Addressing this heterogeneity requires reliable methods for isolation and characterization. Although variability complicates research, it also provides opportunities for new diagnostic and therapeutic applications^[40]^.

### Separation and identification of EVs

EV isolation remains technically challenging, and current extraction methods include ultracentrifugation (UC), sedimentation, ultrafiltration (UF), affinity separation, size-exclusion chromatography (SEC)^[41]^ and density gradient centrifugation (DGC)^[42]^. The advantages and disadvantages of each of these methods make it difficult to satisfy the requirements of high purity, high recovery, and ease of operation simultaneously. Separation methods and technical parameters employed across various laboratories exhibit considerable inconsistency^[43]^. Variations in parameters such as centrifugal speed, duration, and temperature during UC can result in differing recoveries and purities of EVs. Establishing consistent methods to separate EVs is still a major challenge in current studies.

Current methods are not sensitive enough to detect small EVs (sEVs)^[44]^, and nanoparticle tracking lacks precision, creating an urgent need for more advanced detection with higher sensitivity and resolution. The connection between EV composition and function is still unclear^[45]^, so combining different characterization methods with functional studies is necessary to better understand their biological roles.

Optimizing separation and purification techniques, such as density gradient UC, can significantly enhance the purity of EVs while reducing contaminations, such as lipoproteins. The emergence of single-EV analysis techniques, including nano-flow cytometry and single-particle dark-field imaging, enables researchers to analyze the heterogeneity of EVs with greater precision. The diversity within EV populations makes it necessary to create standardized functional assays that provide consistent ways to evaluate and compare their subtypes in biomedical settings. Although isolating, purifying, and characterizing EVs remains difficult, ongoing technological progress is expected to mitigate these barriers and expand their use in both diagnostics and therapy.

### Engineering of EVs

While the engineering of EVs shows significant promise, these entities encounter numerous challenges related to drug-loading efficiency, targeting enhancement, and the preservation of natural properties. Thus, urgent breakthroughs are necessary to achieve their widespread application in disease treatment. The engineering techniques used to modify EVs primarily encompass two approaches: altering the parental material to produce therapeutic EVs and modifying EVs post-isolation^[46]^. Natural EVs are derived from unmodified parent cells, while engineered EVs can originate from both modified parental sources and naturally occurring EVs^[47]^. The efficiency of drug-carrying and targeting mechanisms remains a paramount challenge in EV engineering. Methods such as electroporation, sonication, and chemical transfection can load therapeutic molecules into EVs but may damage vesicle integrity and weaken biological function^[48]^. Engineering strategies have been developed to enhance the targeting efficiency of EVs, which improves drug delivery and changes their natural properties, influencing distribution and activity *in vivo*. Engineered EVs are viewed as promising tools for therapy, drug transport, and diagnostics^[17]^.

### Histologic analysis and mechanistic studies of EVs

Understanding EV mechanisms remains difficult^[49]^. Uptake and signaling seen *in vitro* often differ from their roles *in vivo* because physiological factors such as blood flow and immune activity affect EV behavior^[50,51]^. The processes of formation and internalization are not fully explained, and the biogenesis and release of subtypes like exosomes and microvesicles are still debated^[37]^. How proteins, RNA and lipids are selectively loaded into EVs is unclear, limiting knowledge of their functions^[52]^. Their dynamic behavior, targeting, and interactions with other cells in complex environments also remain poorly understood.

Despite persistent fundamental challenges in EV research - including subtype heterogeneity, unclear mechanisms of generation and release, and complexities in cargo-loading regulation - traditional experimental approaches have reached a significant bottleneck. Concurrently, emerging interdisciplinary paradigms are revitalizing the field. AI is redefining the boundaries of life science research through its robust capabilities in data integration, pattern analysis, and dynamic modeling. By employing machine learning to interpret high-throughput histological data, utilizing deep learning to simulate EV biogenesis mechanisms, and leveraging generative AI to predict unknown biological pathways, researchers are positioned to overcome current research dilemmas. These innovative AI advancements represent a pivotal breakthrough in addressing these challenges, heralding new transformative potential for EV research.

## OVERVIEW OF AI

AI is a technological system that emulates human cognition through algorithms, with core capabilities encompassing data-driven pattern recognition, complex system modeling, and multimodal knowledge fusion. Cutting-edge methodologies such as machine learning, deep learning, and generative AI are overcoming the empirical limitations of traditional life science research, particularly in high-dimensional data analysis, dynamic mechanism deduction, and cross-scale correlation mining, thereby demonstrating transformative potential. These capabilities offer a novel pathway for achieving systematic breakthroughs in the bottlenecks of EV research.

### Machine learning

AI is a branch of computer science that builds systems imitating human cognition including learning and problem solving^[53]^. Machine learning is a core part of AI that analyzes biological data to generate precise predictions of patterns^[53-55]^. With advances in biotechnology and the growing size and complexity of biological datasets, the use of machine learning in biological research is expanding steadily. Machine learning enhances data analysis and model creation in biological research, offering a range of techniques to tailor to different datasets effectively. Through machine learning, vast datasets are inputted, and the computer uses algorithms to discern underlying physical laws, whether known or yet undiscovered, without direct human intervention. Unlike traditional computers that rely on coded algorithms provided by humans, operating like calculators executing predefined commands, machine learning constructs predictive models based on incomplete data sets from databases^[54]^.

#### Types of machine learning

**Figure 1:**
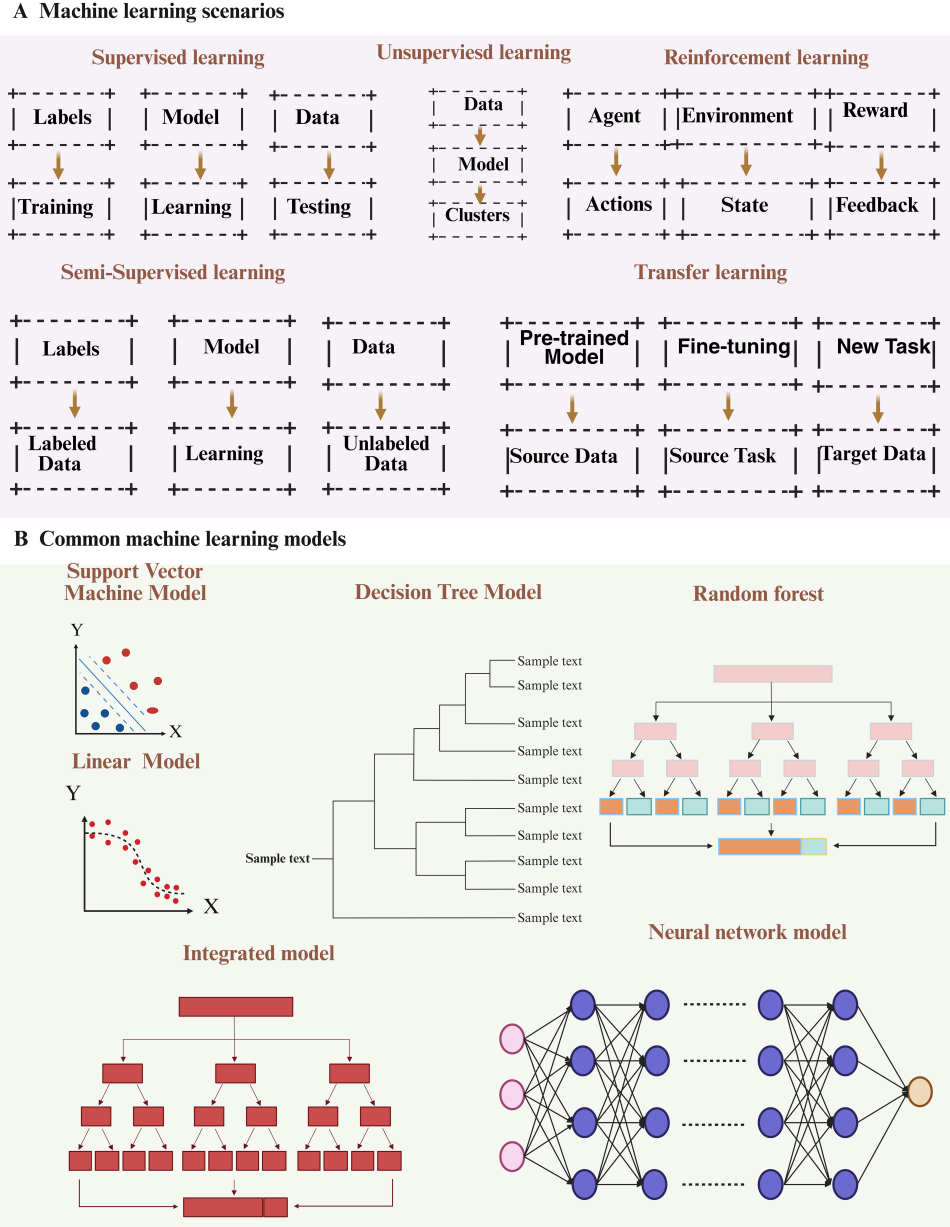
A concise overview of machine learning. (A) Diagram illustrating the five categories of machine learning; (B) Typical machine learning algorithms. Figure was created with https://app.biorender.com/.

Machine learning is a computational approach that derives patterns from data to support decision making, with main categories of supervised, unsupervised, semi-supervised, reinforcement (RL), and transfer learning [Figure 1]. These methods allow machines to continuously enhance their performance and improve the accuracy of their predictions^[56]^. Furthermore, effective post-processing can be achieved by leveraging the robust data processing capabilities of machine learning.

Supervised learning

Supervised learning uses labeled data to train algorithms that classify inputs or predict outcomes through predefined labels. Throughout the training phase, a dataset with answers (labels or targets) is utilized to guide the learning process^[57]^. For instance, supervised learning algorithms can be trained on established disease datasets to anticipate disease types or trends in new cases. By examining patients’ genomic, clinical, and epidemiological data, it becomes feasible to predict potential illnesses or forecast disease progression.

Unsupervised learning

 Unlike supervised learning, unsupervised learning does not depend on labeled data and is unable to measure effects; instead, it relies entirely on the inherent structure of the data. This training method lacks a predefined objective and cannot predict future outcomes^[57,58]^. The primary goal is to extract knowledge through pattern identification. A major limitation of unsupervised learning lies in the difficulty of evaluating model quality, since a definitive ground truth is not available^[57]^. Nevertheless, this approach can be applied to annotate complex, multidimensional datasets, which can then be further analyzed using supervised learning methods to achieve deeper insights. A representative example is single-cell transcriptome sequencing, where unsupervised learning is used to group cells into clusters based on similarities in their transcriptional profiles^[59]^. Unsupervised learning has been applied effectively to DNA microarray data; the fundamental idea involves checking whether each individual possesses a specific gene based on gene expression levels.

RL

 RL is fundamentally distinct from supervised and unsupervised learning approaches, as it does not rely on artificially generated training data, but rather learns through a process of trial and error. RL aims to solve decision-making problems based on the principle of reward and punishment. Unlike supervised learning, RL does not have explicit answers or labels; instead, it seeks to maximize cumulative rewards by interacting with the environment and gaining experience through mistakes^[60]^. While RL holds great potential in the biomedical domain, it still faces significant obstacles in practical application. In practice, RL could be leveraged for personalized medical customization, providing patients with optimal treatment plans and drug dosages by analyzing their genome, lifestyle factors, and other relevant data.

Semi-supervised learning

 Semi-supervised learning combines supervised and unsupervised methods by training models with both labeled and unlabeled data^[61]^. Semi-supervised learning overcomes this limitation by expanding the training set and improving learning performance under constrained labeled data, leveraging both labeled and unlabeled data. For instance, semi-supervised learning can be applied to cancer diagnosis by collecting pathological images of cancer patients, including labeled images and unlabeled images. Feature extraction can then be performed on the pathological images, such as through deep learning techniques. Subsequently, a model is constructed using semi-supervised learning algorithms and trained on the labeled and unlabeled images, enabling the model to learn the features that distinguish between cancer and non-cancer. The trained model is then evaluated on a test set, and its accuracy, recall rate, and other performance indicators are calculated. Finally, the model is optimized based on the evaluation results to enhance its classification performance.

Transfer learning

 Transfer learning emphasizes leveraging labeled or unlabeled data to develop predictive models for future data prediction^[62]^. It involves utilizing knowledge from domain A to aid in building a model for domain B, ultimately enhancing the generalization performance of the latter. For instance, when transitioning from a trained model for recognizing handwritten digits to recognizing new handwritten digits, this process is referred to as migration learning. Migration learning reduces reliance on source domain data, mitigates issues stemming from inconsistent data distribution between domains, and minimizes target domain deviation. Additionally, leveraging pre-trained models in migration learning decreases training costs and expedites training efficiency.

**Figure 2:**
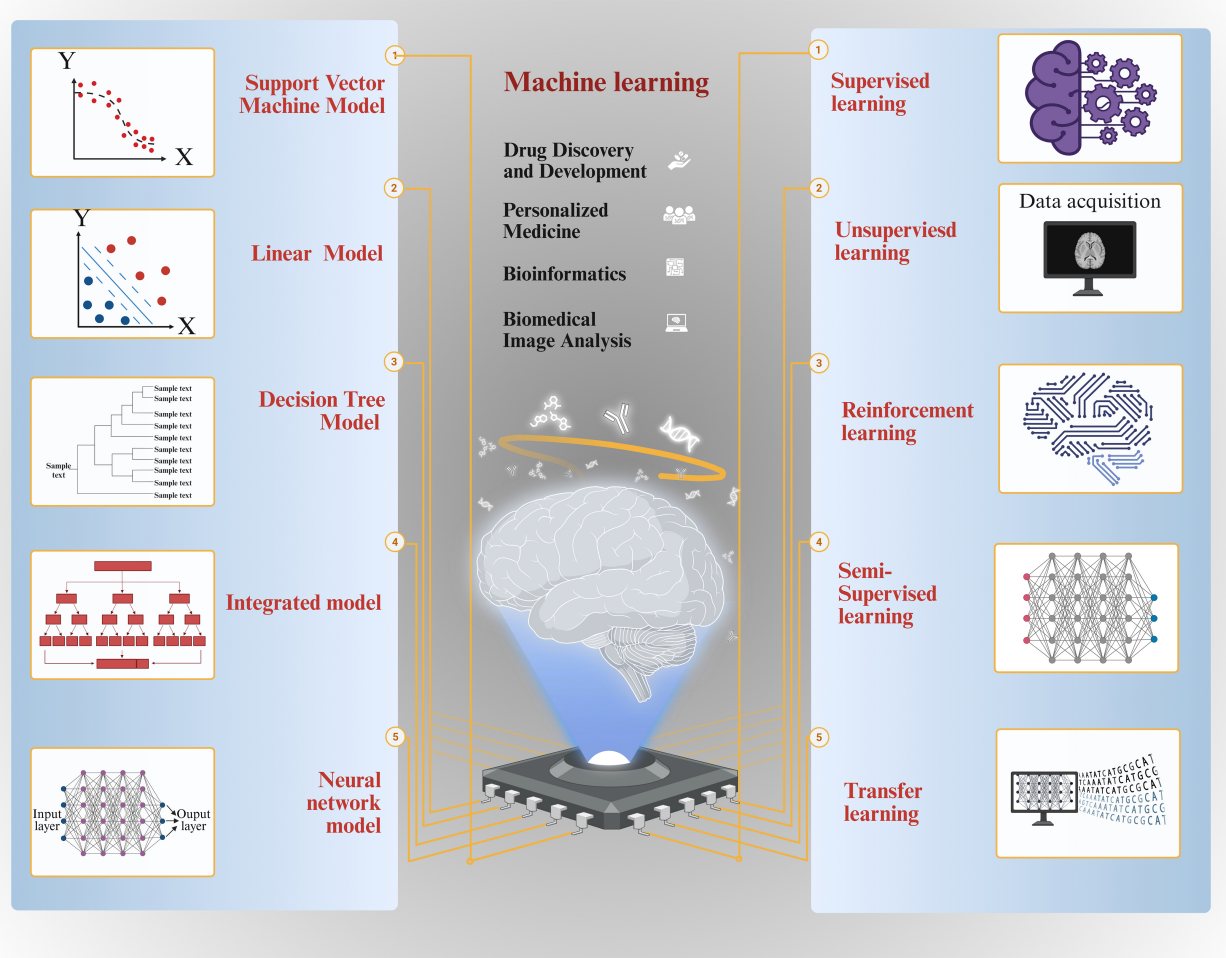
A concise overview of machine learning. (A) Diagram illustrating the five categories of machine learning; (B) Typical machine learning algorithms. Figure was created with https://app.biorender.com/.

Machine learning provides a diverse array of tools and methods that can be combined to tackle complex, real-world problems. Many sophisticated machine learning systems employ multiple approaches to enhance the accuracy and efficiency of decision making, drive technological innovation, and enable substantial advancements across a wide range of fields [Figure 2]. Beyond classical machine learning, emerging quantum machine learning (QML) models demonstrate transformative potential in EV research. By leveraging quantum parallelism, QML algorithms can analyze the high-dimensional electrokinetic properties of EVs with minimal training data - addressing a key limitation of classical machine learning in resource-constrained scenarios. For instance, Thakur *et al*. achieved 71% accuracy in classifying EVs derived from cancer versus non-cancer cells using only 0.5% of the training dataset required by classical models, solely based on electrokinetic signatures^[63]^. This capability is particularly valuable for rapid EV subtype identification in liquid biopsies, where sample volumes are limited. Furthermore, QML’s ability to model complex molecular interactions within EVs could accelerate the design of engineered EVs for targeted therapy.

#### Common models of machine learning

In the realm of machine learning, various models are extensively employed for diverse tasks. Support vector machines (SVMs) are particularly effective for small to medium sample sizes and nonlinear regression predictions, utilizing maximum margin classification and high-dimensional spatial mapping techniques. Linear models are characterized by their simplicity and efficiency, while decision tree models construct tree-like structures through recursive data partitioning. Ensemble models enhance performance by integrating multiple individual models, and neural network models establish intricate mapping relationships inspired by biological neural systems. Each of these models possesses distinct advantages and is appropriate for varying data types and task requirements, thus offering a comprehensive range of options for practitioners in the field of machine learning.

SVM

 SVMs classify data by building a hyperplane in a high-dimensional feature space that maximizes the separation between classes^[64]^. They are applied in fields such as speech recognition, text analysis, and time-series prediction^[65]^. Compared with many other algorithms, SVMs provide high predictive accuracy and efficient computation, making them suitable for nonlinear regression and for datasets of limited or moderate size^[66]^. The main forms of SVMs are linear and nonlinear models.

Linear models

 Linear models represent the most fundamental class of models, which divide or predict data using a straight line or hyperplane. Common examples of linear models include linear regression^[67]^ and logistic regression.

Decision tree Decision tree models are based on a tree structure that recursively divides a dataset into smaller subsets until each subset contains a single class of data points. Common types of decision tree models include classification trees and regression trees^[68,69]^.

Integrated models

 Integrated models are a class of models that enhance predictive performance by combining multiple individual models. Common examples of integrated models include random forest (RF) Integrated models: integrated models are models that improve prediction performance by combining multiple models. Common integration models include RF^[70]^ and Gradient Boosting Tree^[71]^.

Neural network

 Neural network models are based on the biological nervous system and create intricate mapping relationships through connections between multiple neurons^[72,73]^. Typical neural network models encompass multilayer perceptrons, convolutional neural networks (CNNs)^[74,75]^, and recurrent neural networks^[76]^ [Figure 2]. The diversity and flexibility of these models offer a myriad of options for addressing various machine learning problems, as well as a robust foundation for future research and applications.

#### Neural networks and deep learning

The preceding discussion focuses on the classical domain of machine learning. Artificial neural networks (ANNs) derive their name from the objective of modeling interconnected biological neurons. Central to deep learning, ANNs have experienced a resurgence in recent decades, following their initial proposal many years ago. A neural network, a type of machine learning model, emulates a biological nervous system through its multiple neurons, which enhance performance via training and learning^[77]^. ANNs were initially proposed decades ago, but have recently experienced a resurgence in momentum. A neural network is a machine learning model that emulates a biological nervous system through its multiple neurons, which enhance performance via training and learning. These versatile networks can be applied in a variety of fields, including classification, prediction, and pattern recognition. ANNs utilize diverse algorithms to simulate specific functions of the human brain and possess the capacity to learn from experience. Akin to the human brain, ANNs exhibit significant advantages. Consequently, ANNs are employed to address complex problems such as pattern classification, polymer classification, and optimization.

Deep learning is a part of neural network research that studies deep neural networks (DNNs), models defined by having multiple hidden layers. These models can automatically extract hierarchical feature representations from raw data by stacking several nonlinear transformation layers. For instance, in image recognition, deep CNNs automatically extract features such as edges, textures, and shapes from pixel data and subsequently classify these features. Its robust feature learning capability and effective model representation make it a prominent area of research within AI.

### The interplay among AI, machine learning, and deep learning

AI includes a range of methods and technologies that allow computers to carry out tasks similar to human intelligence. Its main purpose is to build systems that reproduce cognitive functions and solve complex problems. Machine learning enables computers to improve by finding patterns in data and creating models that learn from training sets to make predictions or decisions. Deep learning, as a branch of machine learning, uses multilayer neural networks to detect complex features, process raw data directly, and achieve strong performance in many applications. The growth of deep learning has been supported by large datasets, powerful computing resources, and advances in algorithms.

**Figure 3:**
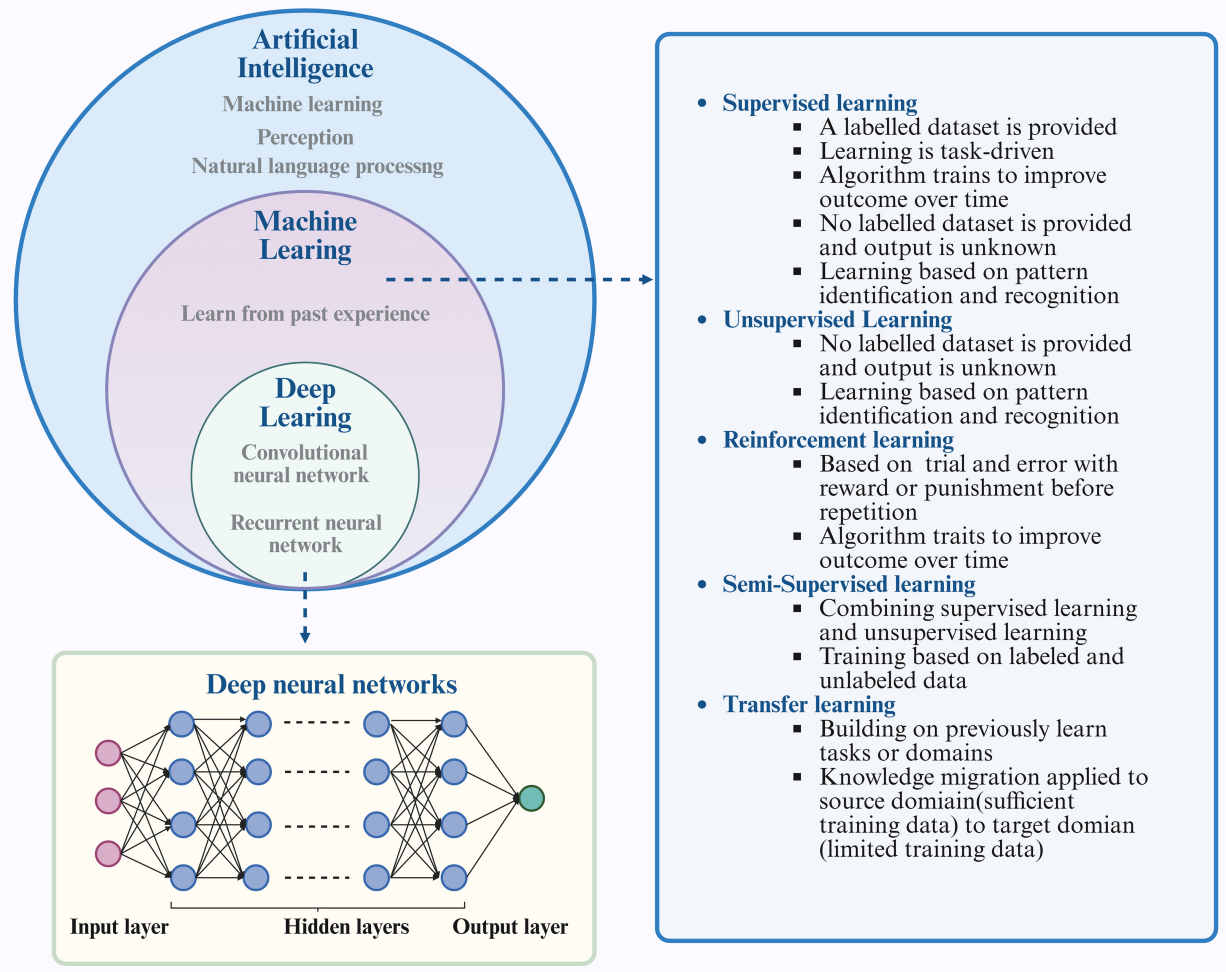
The interplay among AI, machine learning, and deep learning. Machine learning is a fundamental technological approach within the realm of AI, encompassing various methodologies such as supervised learning, unsupervised learning, semi-supervised learning, reinforcement learning, and transfer learning. Deep learning, a branch of machine learning, centers on the use of ANNs to replicate the architecture and operations of the human brain. Figure was created with https://app.biorender.com/. AI: Artificial Intelligence; ANNs: artificial neural networks.

Consequently, these fields can be viewed as a hierarchical structure. AI represents the broader concept, with machine learning being a sub-field within AI. Furthermore, Deep learning is a specific method within the domain of machine learning [Figure 3].

## AI EMPOWERING EVs

The future of AI-mediated engineered EVs holds promise in several key aspects. Firstly, AI technologies are poised to significantly expedite the design and optimization processes of EVs. Through the utilization of big data and machine learning algorithms, AI can swiftly discern intricate relationships and potential patterns within organisms, thereby guiding the development of more functional and efficient EVs. This capability enables researchers to achieve precise organismal modifications promptly, thereby catalyzing innovation across various application domains. Secondly, AI applications enhance the intelligence and efficiency of EV engineering. AI facilitates the automation of experimental design, data collection, and analysis, mitigating errors and reducing the time-intensive nature of manual operations. Consequently, research efficiency and accuracy are heightened, allowing researchers to concentrate on innovative experimental designs and comprehensive data interpretation. This advancement accelerates the development and proliferation of novel biological products. Moreover, AI technologies address challenges inherent in EV design processes. By simulating and forecasting organismal behaviors, AI aids in comprehending the impacts of genetic editing on organisms and offers optimized solutions to mitigate potential risks and side effects. This ensures enhanced safety and control of EVs, thereby promoting their widespread application in medicine, industry, and environmental sectors.

**Figure 4:**
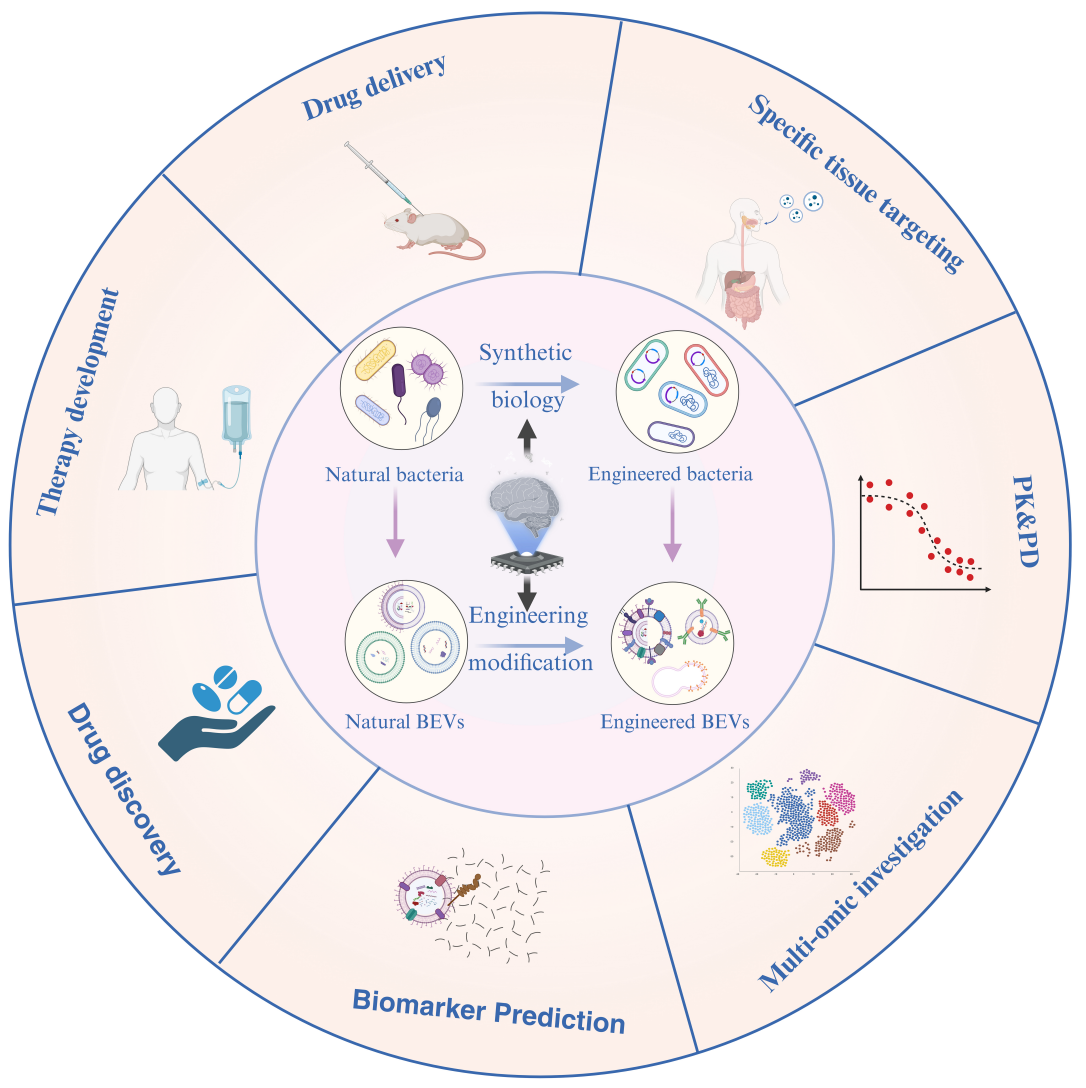
Applications of AI-enabled engineered BEVs. Natural bacteria can be engineered through synthetic biology to create customized strains for the production of tailored BEVs. Alternatively, naturally occurring BEVs can be enhanced using various engineering techniques to develop modified versions. However, AI can direct both natural and engineered BEVs that can be effective in multiple ways. Figure was created with https://app.biorender.com/. AI: Artificial intelligence; PK/PD: pharmacokinetics /pharmacodynamics; BEVs: bacterial extracellular vesicles.

The future prospects of AI-driven EVs are highly promising. As AI technology continues to advance and find practical applications, we can anticipate the emergence of increasingly innovative biological products and solutions, which hold the potential to deliver substantial benefits and new possibilities to human society. EVs secreted by cells are primarily composed of phospholipid bilayers and contain relatively few organelle structures. In recent years, the utilization of EVs for drug delivery has gained significant popularity. Furthermore, AI significantly contributes to the advancement of drug delivery research concerning EVs [Figure 4].

The application of AI in targeted delivery of EVs is primarily reflected in the following key aspects: targeted recognition and selective delivery, engineering strategies, mapping of cellular communication networks for precision treatment, and route optimization and control. A comprehensive examination of these dimensions provides a framework to thoroughly explore the potential of AI in this domain, thereby promoting the optimization and advancement of AI-enabled EV applications.

### Harnessing AI for isolation and characterization of EVs

**Figure 5:**
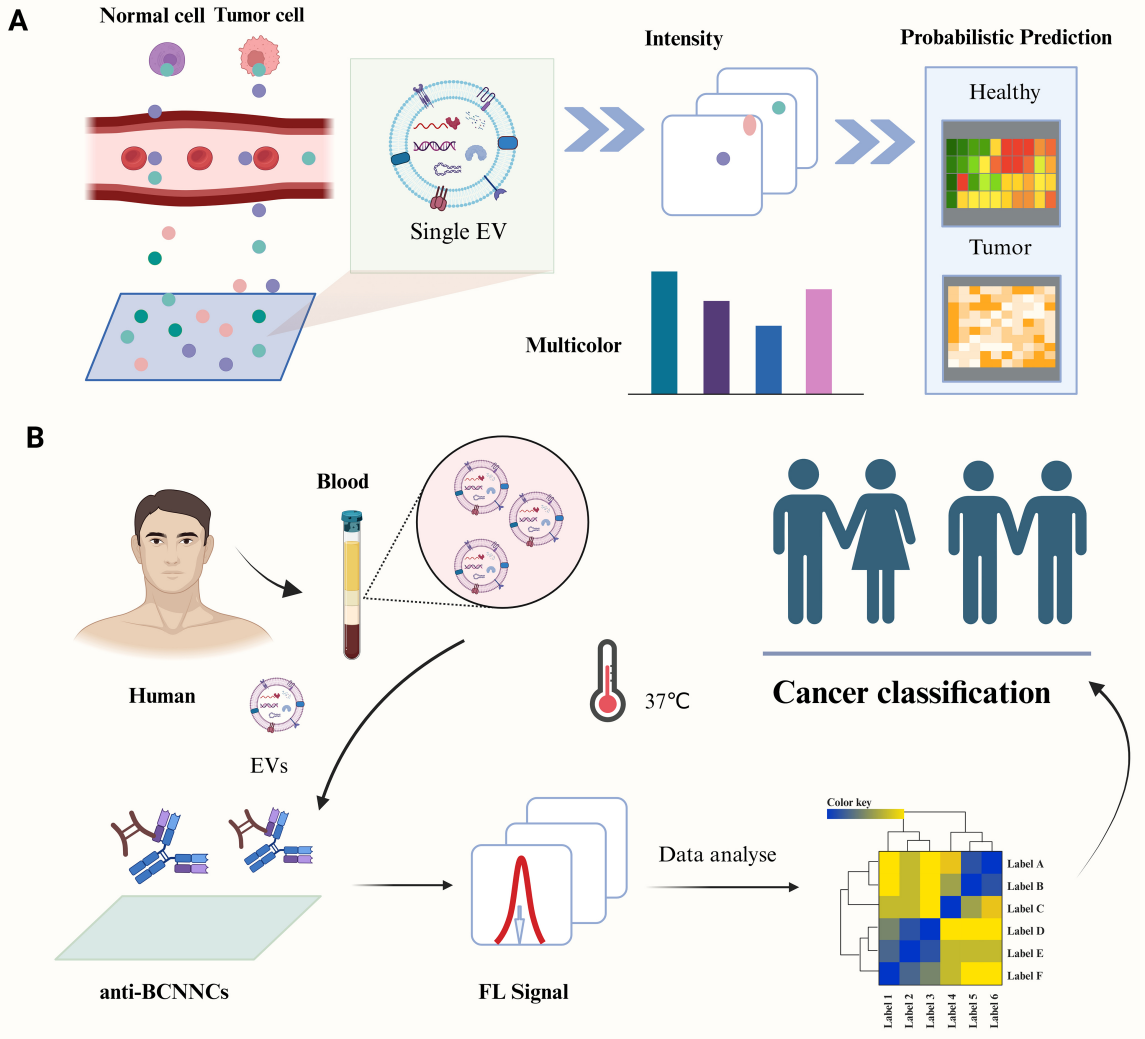
Harnessing AI for isolation of EVs. (A) Employing a sandwich immunoassay alongside KNN and SVM techniques for the specific capture and visualization of individual sEVs; (B) An improved fluorescence-based method for the isolation and detection of sEVs, combined with LDA for analytical purposes. Figure was created with https://app.biorender.com/. AI: Artificial intelligence; EVs: extracellular vesicles; LDA: Linear discriminant analysis; sEVs: small extracellular vesicles; SVM: support vector machine; KNN: K-nearest neighbors.

AI is increasingly applied in EV isolation and analysis, leading to important advances in the field^[78]^. Xiao *et al*. designed a probabilistic system using single-molecule multicolor imaging to capture individual EVs and identify their cellular origin [Figure 5A]^[79]^. Using the immunoaffinity of boric acid-directed coupling, Wang *et al*. utilized an immunoaffinity-based boric acid-directed coupling to develop a 37-min integrated separation-detection scheme for sensitive analysis of EVs [Figure 5B]^[80]^. Furthermore, machine learning and deep learning can be integrated with state-of-the-art nanostructured sensors to identify EVs, enabling efficient processing of heterogeneous EV data and more granular analysis than traditional analytical methods^[81]^. In addition, Jalali *et al*. designed a multifluidic platform based on surface-enhanced Raman scattering, incorporating an array of nanocavity microchips to enable early detection and monitoring of glioblastoma-derived sEVs, with sensitivity reaching the level of a single vesicle^[82]^.

AI technology demonstrates significant potential in the isolation and characterization of EVs. Through the integration of intelligent probabilistic systems, immunoaffinity separation, machine learning coupled with nanosensors, and Raman scattering-based multifluidic devices, researchers have achieved efficient capture, precise imaging, and single-molecule-level traceability of EVs, thereby significantly enhancing analytical sensitivity and resolution. These advancements provide robust tools for the application of EVs in disease diagnosis, therapeutic monitoring, and biomarker discovery, heralding a promising future for precision medicine and personalized therapy.

### Harnessing AI for targeted recognition and selective delivery of EVs

AI can characterize and analyze EVs through algorithms such as machine learning and deep learning to identify and select specific targets, such as tumor cells or inflammatory sites. Subsequently, appropriate targeting strategies can be developed to ensure precise interaction between the vesicles and target cells.

In recent years, the utilization of EVs for drug delivery has gained significant popularity. Furthermore, AI is essential for enhancing research on the management of EVs. However, targeted drug delivery *in vivo* remains one of the most promising methods for the treatment of many challenging and complex diseases, such as tumors, Alzheimer’s disease, and certain bone disorders. Effectively transporting drugs through appropriate carriers to the targeted sites is a key challenge. In the biomedical field, the application of machine learning has been steadily increasing. Jin *et al*. developed a cancer diagnostic method using biomarker detection in body fluids^[83]^ and built a breast cancer liquid biopsy platform that combines a fluorescence sensor array with a deep learning model called AggMapNet. Through the analysis of fluorescence spectrum data, this system can accurately classify different cancer cells, presenting a promising non-invasive approach for breast cancer diagnosis. Furthermore, Shen *et al*. designed a novel unsupervised feature aggregation tool, AggMap, which demonstrated excellent feature reconstruction capabilities when dealing with random benchmark datasets^[84]^. The unsupervised AggMap algorithm leverages its exceptional feature reconstruction capabilities by integrating supervised and interpretable AggMapNet architectures, thereby establishing a system that improves the learning and interpretation of low-sample histological data.

Targeted identification represents the initial crucial step; the challenge lies in ensuring the specific delivery of EVs to precise sites and their uptake by specific cells. Current methods for accurately targeting EVs may yield undesired effects. Utilizing AI can aid in developing novel targeting ligands that mitigate the drawbacks of conventional methods, enabling accurate and swift localization of these ligands to target EVs without error. In comparison to other synthetic carriers, EVs offer distinct advantages, including a safe source, enhanced biocompatibility, and patient tolerance. Furthermore, EVs are abundantly present and easily accessible in the human body. Through screening and functional enrichment analysis of EV phenotypes with the aforementioned techniques, precise drug delivery via EV carriers can be achieved. The system will consider individual and environmental factors, programming EVs to release drugs continuously over an extended period to achieve sustained therapeutic effects with AI assistance. AI can analyze and store patient databases to tailor the best treatment according to individual conditions, thus yielding favorable treatment outcomes. This strategy improves patient comfort, reduces risk, increases accuracy, enhances treatment effectiveness, and shortens therapy duration. Integrating AI with EV-based drug delivery systems may reshape conventional methods and support the development of personalized medicine.

### Harnessing AI for the engineering of EVs

**Figure 6:**
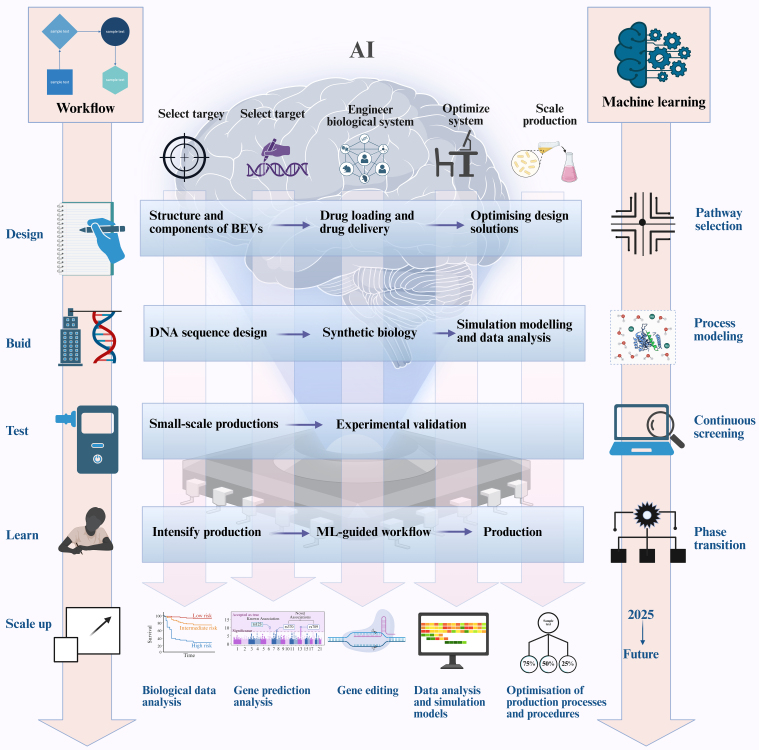
AI-powered synthetic biology for the production of BEVs using a design-build-test-learn cycle. The integration of AI can improve the efficiency of the design-build-test-learning cycle, enabling more effective use of synthetic biology to produce EVs. Figure was created with https://app.biorender.com/. AI: Artificial intelligence; BEVs: bacterial extracellular vesicles; EVs: extracellular vesicles.

The integration of AI with EV engineering is revolutionizing therapeutic development by overcoming persistent translational barriers. The design-build-test-learn (DBTL) cycle in EV engineering closely mirrors the iterative processes of machine learning [Figure 6]. In recent years, significant progress has been achieved in applying AI technologies to EV engineering, particularly in addressing critical challenges including scalable manufacturing, standardization, drug-loading efficiency, targeted delivery, safety and immunogenicity, as well as *in vivo* pharmacokinetics and stability.

Traditional EV manufacturing suffers from batch-to-batch variability and low yields. RL algorithms dynamically optimize bioreactor parameters (e.g., pH, shear stress) by simulating 10^6+ culture conditions, achieving > 90% yield consistency across scales^[85]^. CNNs coupled with inline Raman spectroscopy enable real-time quality control, detecting subtle EV heterogeneity (< 5% size variation) that is undetectable by conventional methods^[86]^.

AI demonstrates significant advantages in enhancing the drug-loading efficiency and targeted delivery of EVs. By simulating and analyzing the interactions between EVs and drug molecules, AI enables the optimization of drug-loading efficiency. For instance, deep learning models can predict optimal encapsulation modalities for drug molecules within EVs, thereby improving drug stability and delivery efficacy. Regarding targeted delivery, AI facilitates the design of more effective targeting strategies by analyzing EV surface markers and receptors on target cells. Specifically, machine learning algorithms can identify high-affinity targeting ligands, substantially enhancing EV enrichment within tumor tissues^[87]^.

AI plays an increasingly critical role in evaluating the safety and immunogenicity of EVs^[88]^. By correlating the biophysical properties of EVs with immunogenicity data, AI enables predictive assessment of EV immunogenicity to guide the design of safer therapeutic EV products. Furthermore, AI facilitates strategic surface engineering to systematically reduce immunogenicity. For instance, Nano-flow cytometry analysis at single-particle resolution allows comprehensive optimization of production conditions to enhance drug-loading yield, while simultaneously evaluating the impact of storage parameters on EV stability and functionality.

AI offers significant advantages in modeling the *in vivo* pharmacokinetics and stability of EVs^[89]^. By developing mathematical models, AI enables the simulation of EV biodistribution, metabolism, and excretion processes. For instance, deep learning architectures can predict tissue-specific accumulation kinetics of EVs, thereby informing rational redesign to enhance their stability. Furthermore, AI facilitates structural and compositional optimization of EVs to improve their *in vivo* persistence.

### Harnessing AI for cellular communication mapping of EVs

Mapping cellular communication is a crucial step in comprehending the interactions among cells within biological systems. By employing a multi-omics approach to EVs, the communication network between cells can be elucidated more comprehensively and accurately. EVs obtained at different stages of a patient’s life can provide valuable information for identifying disease progression and evaluating treatment responses, while multi-omics approaches reveal dynamic molecular alterations underlying disease development^[90-92]^. As an example, Cohn *et al*. demonstrated the role of glial cell-derived EVs in Alzheimer’s disease by applying proteomic and miRNA profiling to microglia-derived vesicles^[90]^. Luo *et al*. investigated the multi-omic characterization of EVs released by human lung adenocarcinoma stem cells, many of which are associated with tumor formation^[91]^. Identifying highly expressed lung adenocarcinoma stem-like cell (LSLC) markers in the EVs produced by these stem cells is critical; these markers may serve as liquid biopsy biomarkers for diagnosing lung adenocarcinoma, potentially making significant contributions to tumor growth and stability. Furthermore, Lam *et al*. investigated the proteomics of EVs from COVID-19 patients across various stages, including pre-symptomatic, hyperinflammatory, subsiding, and recovery phases^[92]^. The research emphasized the impact of disrupted raft lipid metabolism on the lipid membrane alterations in EVs. The integration of EVs data with cellular multi-omics information holds promise for advancing drug discovery, precision therapy, and biomolecular applications.

### Harnessing AI for multi-omics data analysis of EVs

The rapid advancement of high-throughput sequencing technology has ushered in an era of integration in multi-omics research, encompassing a wide range of fields. Analyzing multi-omics data from EVs is crucial for elucidating disease mechanisms and identifying potential biomarkers. Furthermore, the continuous progress in AI technologies, particularly machine learning and deep learning models, offers robust tools for the analysis of multi-omics data related to EVs. The application of AI in multi-omics involves a range of tasks, including classification (such as SVM, graph neural networks (GNNs), and Transformers, which achieve over 90% accuracy in cancer detection), prediction [utilizing DNNs and variational autoencoders (VAEs) to improve the accuracy of drug response forecasts], generation [employing generative adversarial networks (GANs) and VAEs to mitigate data sparsity], and clustering (utilizing self-coders optimized for subtype classification). Moreover, AI promotes the development of transformer-based models and introduces a novel paradigm for cross-modal integration and dynamic mechanism analysis in precision medicine^[93]^.

One of the challenges in EV research is dealing with the processing of high-throughput multi-omics data. Analyzing such data necessitates the integration of statistical methods with computational modeling. Moreover, the transition of data types from conventional structured formats to unstructured, semi-structured, and heterogeneous architectures presents a further complexity. The interplay among omics data is intricate, involving both linear and nonlinear relationships. Yin *et al*. extensively analyzed the proteomic characteristics of serum EVs using 4D-DIA, identified key biomarkers, and developed an efficient RF diagnostic model, achieving areas under the curve (AUC) of 0.960 in the training set and 0.963 in the test set^[94]^. This model accurately identifies early colorectal cancer (CRC) and distinguishes between CRC and benign lesions, a finding validated through enzyme-linked immunosorbent assay (ELISA) and further assessed via multi-omics functional traceability. Following ELISA validation and multi-omics functional traceability analysis, the model presents a promising EV proteomics solution for the non-invasive diagnosis of CRC, demonstrating high sensitivity and substantial potential for clinical application. By integrating multi-omics data, including genomics, proteomics, and metabolomics, with machine learning techniques, we systematically analyze complex biological interaction networks and identify predictive markers for diseases. This approach offers a novel data-driven paradigm for accurate patient stratification and personalized treatment^[95]^.

### Harnessing AI for disease diagnosis and treatment of EVs

AI holds significant application potential in the discovery of biomarkers associated with EVs^[96,97]^. It can efficiently process and analyze vast quantities of EVs-related biomedical data, and through the application of machine learning and deep learning algorithms, extract biomarkers of potential diagnostic value from complex proteomic, nucleic acidomic, and other multi-omics data. Zhou *et al*. utilized a three-dimensional microfluidic platform integrating quantum dot labeling with vesicle fusion to achieve *in situ* detection of multiple sEV biomarkers^[98]^.

**Figure 7:**
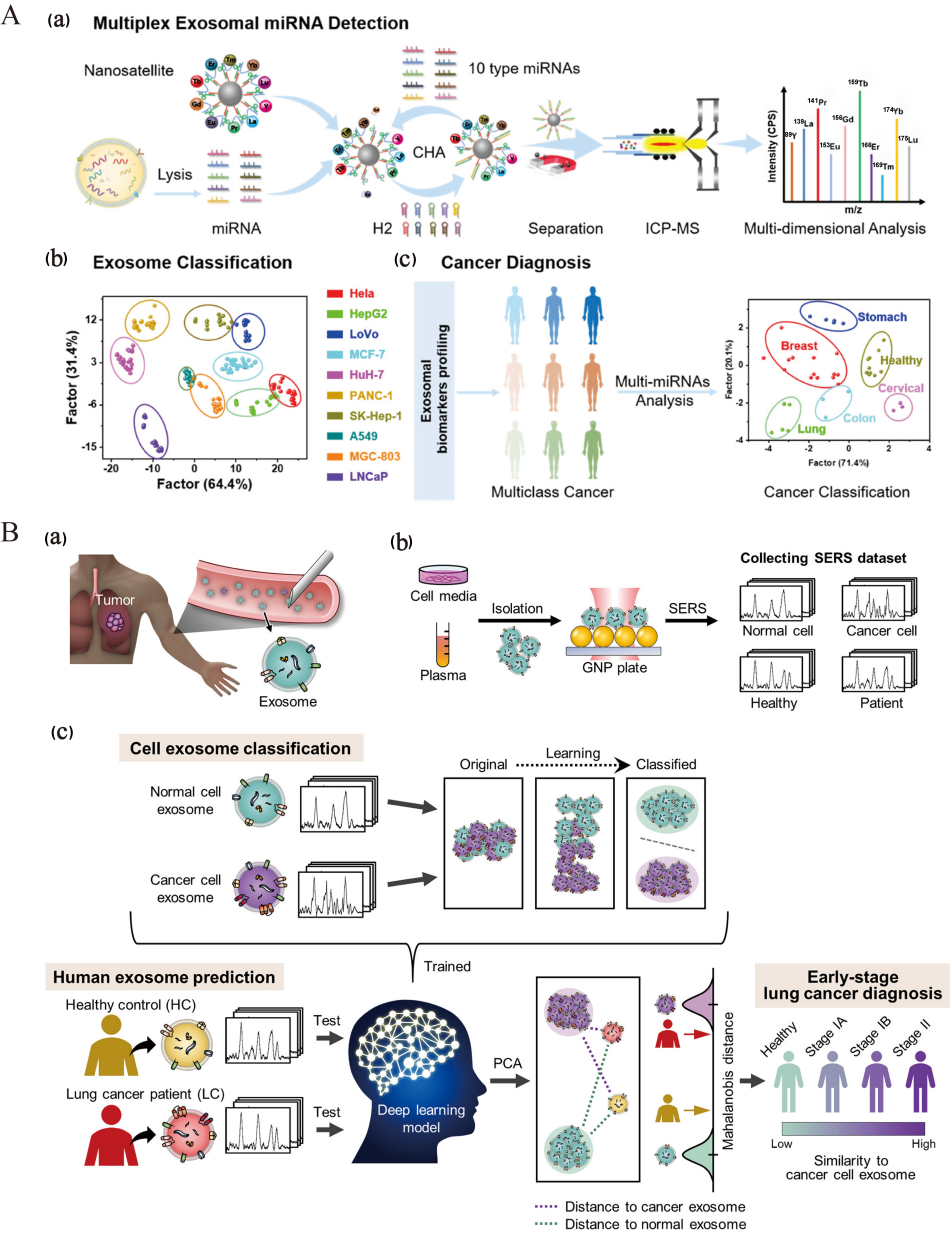
Harnessing AI for disease diagnosis and treatment of EVs. (A) Nanosatellite systems for EV classification and multicancer diagnosis through multiplex detection of miRNAs in EVs^[99]^. Copyright © 2022 American Chemical Society; (B) Schematic representation of deep learning-based analysis of circulating exosomes for lung cancer diagnosis^[100]^. Copyright © 2020 American Chemical Society. AI: Artificial intelligence; EVs: extracellular vesicles; SERS: surface-enhanced Raman scattering.

AI will continue to enhance the application of EVs in disease diagnosis and treatment. Through AI-driven multi-omics analyses and machine learning algorithms, researchers can identify biomarkers and optimize treatment strategies with increased efficacy. Wen *et al*. developed an ultra-multiplex and highly sensitive miRNA analysis method based on ICP-MS for the simultaneous detection of 10 miRNAs in EVs for cancer diagnosis [Figure 7A]^[99]^. Shin *et al*. employed surface-enhanced Raman scattering and deep learning techniques to model the correlation between cells of varying origins and sEVs in plasma, thereby aiding in the prediction of a patient’s risk of developing early-stage lung cancer [Figure 7B]^[100]^. Yang *et al*. developed ChatExosome, an interactive diagnostic system for EVs that leverages a large language model (LLM)^[101]^. The feature fusion transformer (FFT) component of this system enhances the LLM’s ability to analyze the Raman spectra of EVs. Subsequently, a retrieval-augmented generation (RAG) framework is employed to bolster the reliability of the LLM in diagnosing diseases associated with EVs. As AI technology continues to advance, its applications in EV research are expected to expand significantly. Future AI algorithms have the potential to develop smarter automated platforms for the separation and identification of EVs, thereby enhancing the efficiency and accuracy of research in this area. Additionally, leveraging AI technology to explore the mechanisms by which EVs contribute to disease progression further supports their application as liquid biopsy markers, offering new strategies for the early diagnosis and treatment of various diseases^[102,103]^. In conclusion, AI technology presents promising opportunities for EV research and is anticipated to facilitate its widespread application in the biomedical field.

## MAJOR CHALLENGES AND SOLUTIONS OF AI

### Lack of standardization

The standardization of AI is essential in the context of rapidly increasing data volumes, as a lack of standardization can result in numerous challenges. To ensure the credibility of machine learning analyses in the life sciences, the repeatability and replicability of models are critical. Laine *et al*. highlighted a replication crisis in deep learning as evidenced by biological image analysis^[104]^. The performance of machine learning models is contingent on data quality and parameter settings, and their reliability is primarily limited to images that closely resemble the training data; the degree of similarity is influenced by various factors. When training data are inadequate, the results generated by the model become unreliable and difficult to detect. Heil *et al*. proposed a set of evaluation criteria that include a bronze standard (disclosing data, models, and code), a silver standard (ensuring that dependencies are downloadable and installable, recording replication details, and setting deterministic random values), and a gold standard (achieving full automation of the analysis process)^[105]^. Despite the relative ease of attaining reproducibility in computational research, factors such as the experimental environment and materials must also be considered. Karimzadeh *et al*. emphasized that effective documentation is crucial for citing and resolving software issues^[106]^. Further guidance on ensuring reproducibility can be found in the existing literature. Additionally, the implementation of AI in clinical and other domains necessitates thorough ethical considerations.

### Ethical issues

AI is increasingly prevalent in the biomedical field, and as its use expands, addressing the ethical implications becomes ever more crucial. However, the privacy challenges associated with AI in healthcare must not be overlooked. The core issue lies in the trade-off between the extensive data collection that AI facilitates and the erosion of patient privacy, creating a paradox. To ensure that technology enhances patient care while safeguarding privacy, the implementation of AI in medicine presents significant challenges regarding the protection of patients’ bodily data and the practical impacts on both healthcare professionals and patients. In the future, AI systems might autonomously categorize patients or prioritize referrals, potentially leading to inequities and raising important ethical concerns^[107]^.

Machine learning holds significant potential in healthcare; however, it also presents complex ethical issues that are only partially addressed during implementation. Despite uncertainties regarding its future impact, a systematic approach supported by an assessment framework enables proactive management of these uncertainties by comprehensively identifying key factors and predicting potential outcomes. Transparency is essential for managing conflicts of interest; therefore, it is crucial to establish transparent and decentralized systems^[108]^. Such systems facilitate shared responsibility for patient safety among all stakeholders involved in clinical algorithms, thereby enhancing the confidence of all parties in the deployment of AI technologies^[109]^.

### Data quality and privacy data

The progress of AI requires both high-quality data and strong privacy protection. Large datasets are essential for AI, but low-quality data reduce accuracy and weaken reliability. With the growing amount of sensitive personal information, privacy protection has become critical and depends on regulatory policies together with technical tools such as anonymization and encryption. Long-term development of AI depends on maintaining data quality throughout its entire lifecycle, combining technological advances with legal oversight, and building safeguards that prevent misuse while allowing for the effective use of data.

### Modeling bias and fairness

**Figure 8:**
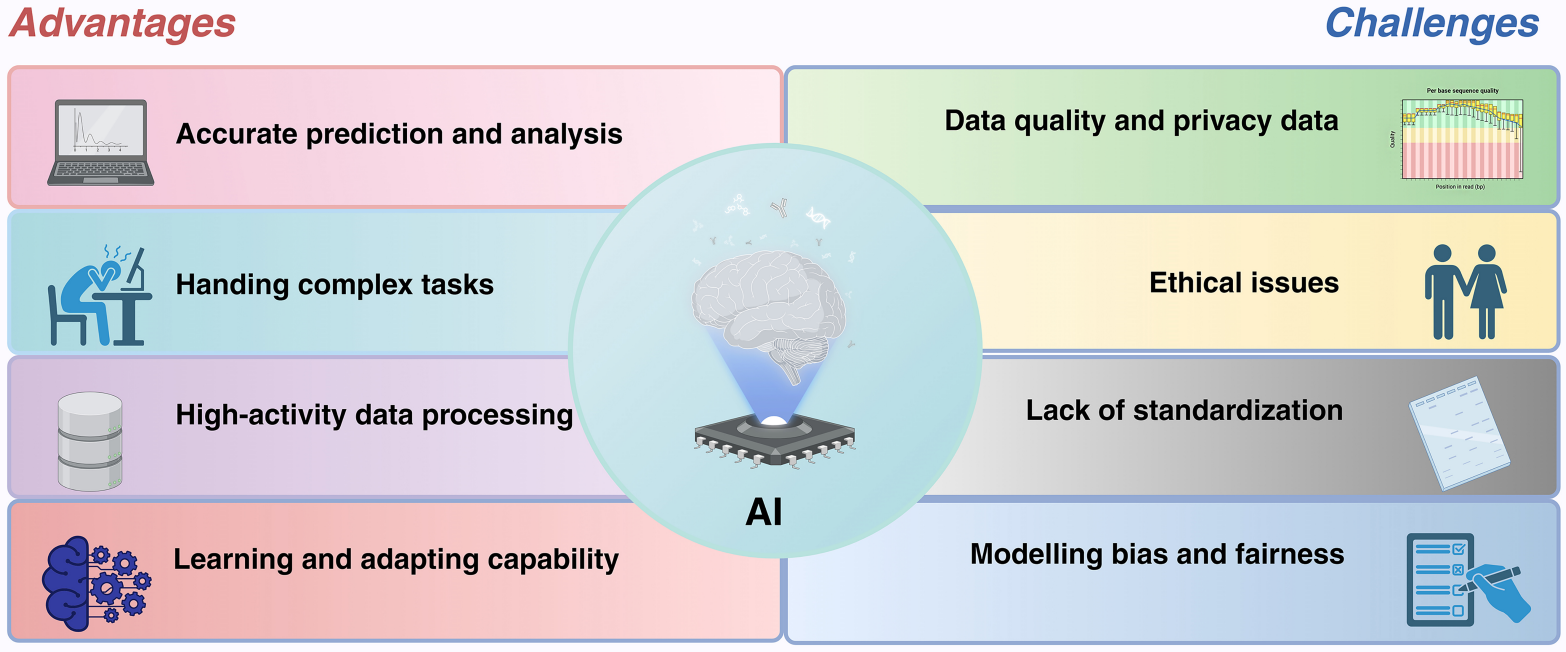
Major advantages and challenges of AI. The integration of AI in EVs offers substantial benefits, including precise prediction and analysis, the capability to handle complex tasks, efficient data processing, and enhanced learning and adaptability. Nevertheless, alongside these advantages, challenges persist, such as ethical concerns, a lack of standardization, data security and privacy issues, and biases concerning model fairness. Figure was created with https://app.biorender.com/. AI: Artificial intelligence; EVs: extracellular vesicles.

AI systems can inherit biases from their training data, leading to unfair results that discriminate against groups defined by race, gender, or other demographic traits. To ensure fairness and neutrality, datasets are carefully examined and adjusted to remove hidden bias, and training data are expanded with diverse and representative samples so that different populations are properly covered without over-relying on any single group. Fairness measures and evaluation methods are applied to check whether outcomes remain balanced across demographic categories. Researchers also design algorithms that reduce group disparities and lower dependence on sensitive features, while improvements in transparency and interpretability make it easier to detect and correct bias, increasing the reliability of automated decisions. Legal and ethical safeguards further require the creation of clear standards and regulations to maintain impartiality. Addressing bias and promoting fairness in AI requires cooperation across fields such as data science, computer science, law, and ethics [Figure 8].

## CONCLUSION AND PERCEPTIVE

This paper provides a comprehensive review of the current status and challenges in EV research, as well as advancements in the application of AI within this domain. The heterogeneity of EVs, along with challenges in their isolation, characterization, engineering, and clinical application, has hindered their widespread adoption in biomedical fields. AI technologies, particularly machine learning and deep learning, offer innovative approaches and tools for EV research. Enhanced by AI, significant progress has been made in target identification of EVs, optimization of drug delivery systems, mapping of cellular communication networks, and analysis of multi-omics data, thereby unlocking new opportunities for the use of EVs as biomarkers and therapeutic vectors. Nonetheless, AI-driven research on EVs encounters several challenges, including a lack of standardization, ethical concerns, data quality and privacy issues, model bias, and fairness.

In the future, AI is expected to drive breakthroughs in EV research. High-resolution imaging and multi-omics analysis will enable comprehensive assessments of EV heterogeneity and functional differences. Furthermore, the development of efficient separation and identification techniques will enhance the purity and recovery rates of EVs, thereby facilitating their clinical applications. For instance, machine learning dynamically recommends optimal operating conditions by analyzing critical parameters (centrifugal force, flow rate) in UC and SEC, thereby reducing vesicle damage^[110]^. Building upon this, microfluidic chips integrated with surface-enhanced Raman scattering (SERS)^[111,112]^ coupled to ResNet architectures enable real-time on-chip detection of single-EV size and surface markers (CD9/63/81), replacing time-consuming manual characterization. Through machine learning algorithms, the differential gradient centrifugation (DGC) protocol can be optimized to ensure that the purity of EVs consistently meets the > 95% standard required by the good manufacturing practice (GMP) regulations^[113]^. Refining the design of EVs is important to enhance their stability and therapeutic function, and clinical trials that verify their use as biomarkers and carriers will expand their medical utility, while the development of consistent standards and ethical rules supported by AI will improve data protection and secure compliance in practice.

AI models, especially those based on deep learning, are frequently regarded as “black boxes” due to their opaque decision-making processes, which restricts their broader use in the clinical translation of EV research. Interpretability can be improved through feature importance analysis that measures the contribution of factors such as CD63 and CD81 in EV targeting, visualization methods that reveal model reasoning by extracting EV features like size distribution and membrane integrity in CNNs, and simplified models that are easier to interpret and provide biologically meaningful rules for clinical validation even with reduced accuracy. Clinical application of AI in EV research is limited by heterogeneity of data from multiple sources requiring standardized collection and processing, the need for multi-center trials to demonstrate safety and efficacy while protecting privacy through methods such as federated learning, and the establishment of regulatory frameworks consistent with medical device certification to support translation into clinical practice.

We plan to develop a ChatGPT-based platform on EVs built on our research foundation, designed for researchers, students, and clinicians, providing explanations of EV biology including concepts, structures, functions, and biomedical applications while presenting recent findings and advances in the field, offering guidance on experimental design, data analysis, and related research activities, supported by a comprehensive database of documents, publications, and datasets to ensure accuracy and reliability, delivering recommendations tailored to user interests through a user-friendly interface that supports text and images, incorporating a feedback system for continuous improvement, and ultimately extending its application to research institutions and educational platforms to increase its impact.

## DECLARATIONS

### Copyright

© The Author(s) 2025.

## References

[1] Liu H, Zhang Q, Wang S, Weng W, Jing Y, Su J (2022). Bacterial extracellular vesicles as bioactive nanocarriers for drug delivery: advances and perspectives. Bioact Mater.

[2] Liu H, Sun J, Wang M, Wang S, Su J, Xu C (2023). Intestinal organoids and organoids extracellular vesicles for inflammatory bowel disease treatment. Chem Eng J.

[3] Han R, Zhou D, Ji N (2025). Folic acid-modified ginger-derived extracellular vesicles for targeted treatment of rheumatoid arthritis by remodeling immune microenvironment via the PI3K-AKT pathway. J Nanobiotechnology.

[4] (2015). Cicero A, Stahl PD, Raposo G. Extracellular vesicles shuffling intercellular messages: for good or for bad. Curr Opin Cell Biol.

[5] Liu H, Su J (2023). Organoid and organoid extracellular vesicles for osteoporotic fractures therapy: current status and future perspectives. Interdiscip Med.

[6] Zhou G, Li R, Sheng S (2024). Organoids and organoid extracellular vesicles-based disease treatment strategies. J Nanobiotechnology.

[7] Théry C, Witwer KW, Aikawa E (2018). Minimal information for studies of extracellular vesicles 2018 (MISEV2018): a position statement of the International Society for Extracellular Vesicles and update of the MISEV2014 guidelines. J Extracell Vesicles.

[8] Wang S, Hu Y, Song P (2025). Harnessing extracellular vesicles from *Lactobacillus reuteri* and *Lactobacillus paracasei* for synergistic osteoporosis therapy. Compos Part B Eng.

[9] Battistelli M, Falcieri E (2020). Apoptotic bodies: particular extracellular vesicles involved in intercellular communication. Biology.

[10] Atkin-Smith GK, Paone S, Zanker DJ (2017). Isolation of cell type-specific apoptotic bodies by fluorescence-activated cell sorting. Sci Rep.

[11] Serrano-Heras G, Díaz-Maroto I, Castro-Robles B (2020). Isolation and quantification of blood apoptotic bodies, a non-invasive tool to evaluate apoptosis in patients with ischemic stroke and neurodegenerative diseases. Biol Proced Online.

[12] Tian Y, Li S, Song J (2014). A doxorubicin delivery platform using engineered natural membrane vesicle exosomes for targeted tumor therapy. Biomaterials.

[13] Kamerkar S, LeBleu VS, Sugimoto H (2017). Exosomes facilitate therapeutic targeting of oncogenic KRAS in pancreatic cancer. Nature.

[14] Alvarez-Erviti L, Seow Y, Yin H, Betts C, Lakhal S, Wood MJ (2011). Delivery of siRNA to the mouse brain by systemic injection of targeted exosomes. Nat Biotechnol.

[15] Batrakova EV, Kim MS (2015). Using exosomes, naturally-equipped nanocarriers, for drug delivery. J Control Release.

[16] Tian T, Zhang HX, He CP (2018). Surface functionalized exosomes as targeted drug delivery vehicles for cerebral ischemia therapy. Biomaterials.

[17] Xu M, Feng T, Liu B (2021). Engineered exosomes: desirable target-tracking characteristics for cerebrovascular and neurodegenerative disease therapies. Theranostics.

[18] Liao Z, Liu H, Ma L (2021). Engineering extracellular vesicles restore the impaired cellular uptake and attenuate intervertebral disc degeneration. ACS Nano.

[19] Gujrati V, Prakash J, Malekzadeh-Najafabadi J (2019). Bioengineered bacterial vesicles as biological nano-heaters for optoacoustic imaging. Nat Commun.

[20] Tran PHL, Xiang D, Tran TTD (2020). Exosomes and nanoengineering: a match made for precision therapeutics. Adv Mater.

[21] Wu P, Zhang B, Ocansey DKW, Xu W, Qian H (2021). Extracellular vesicles: a bright star of nanomedicine. Biomaterials.

[22] Liu H, Li R, Yang H (2025). Extracellular vesicles in gut-bone axis: novel insights and therapeutic opportunities for osteoporosis. Small Sci.

[23] Eisenstein M (2021). Artificial intelligence powers protein-folding predictions. Nature.

[24] Liu H, Triffitt JT, Xia Z, Su J (2024). Artificial intelligence-enabled studies on organoid and organoid extracellular vesicles. Biomater Transl.

[25] Kumar S, Stecher G, Tamura K (2016). MEGA7: molecular evolutionary genetics analysis version 7.0 for bigger datasets. Mol Biol Evol.

[26] Ronquist F, Huelsenbeck JP (2003). MrBayes 3: bayesian phylogenetic inference under mixed models. Bioinformatics.

[27] Saitou N, Nei M (1987). The neighbor-joining method: a new method for reconstructing phylogenetic trees. Mol Biol Evol.

[28] Mortazavi A, Williams BA, McCue K, Schaeffer L, Wold B (2008). Mapping and quantifying mammalian transcriptomes by RNA-Seq. Nat Methods.

[29] Smyth GK (2004). Linear models and empirical bayes methods for assessing differential expression in microarray experiments. Stat Appl Genet Mol Biol.

[30] Wen M, Wang J, Ou Z (2023). Bacterial extracellular vesicles: a position paper by the microbial vesicles task force of the Chinese society for extracellular vesicles. Interdiscip Med.

[31] Liu H, Wu Y, Wang F (2023). Bone-targeted engineered bacterial extracellular vesicles delivering miRNA to treat osteoporosis. Compos Part B Eng.

[32] Ji N, Wang F, Wang M, Zhang W, Liu H, Su J (2023). Engineered bacterial extracellular vesicles for central nervous system diseases. J Control Release.

[33] Liu H, Li M, Zhang T (2022). Engineered bacterial extracellular vesicles for osteoporosis therapy. Chem Eng J.

[34] Liu H, Zhang H, Han Y, Hu Y, Geng Z, Su J (2022). Bacterial extracellular vesicles-based therapeutic strategies for bone and soft tissue tumors therapy. Theranostics.

[35] Wu Y, Song P, Wang M, Liu H, Jing Y, Su J (2024). Extracellular derivatives for bone metabolism. J Adv Res.

[36] Raposo G, Stahl PD (2023). Extracellular vesicles - on the cusp of a new language in the biological sciences. Extracell Vesicles Circ Nucl Acids.

[37] Kowal J, Arras G, Colombo M (2016). Proteomic comparison defines novel markers to characterize heterogeneous populations of extracellular vesicle subtypes. Proc Natl Acad Sci U S A.

[38] (2018). van Niel G, D’Angelo G, Raposo G. Shedding light on the cell biology of extracellular vesicles. Nat Rev Mol Cell Biol.

[39] Peinado H, Alečković M, Lavotshkin S (2012). Melanoma exosomes educate bone marrow progenitor cells toward a pro-metastatic phenotype through MET. Nat Med.

[40] Jiang C, Fu Y, Liu G, Shu B, Davis J, Tofaris GK (2021). Multiplexed profiling of extracellular vesicles for biomarker development. Nanomicro Lett.

[41] Bracht JWP, Los M, van der Pol E (2024). Choice of size-exclusion chromatography column affects recovery, purity, and miRNA cargo analysis of extracellular vesicles from human plasma. Extracell Vesicles Circ Nucl Acids.

[42] Shami-Shah A, Travis BG, Walt DR (2023). Advances in extracellular vesicle isolation methods: a path towards cell-type specific EV isolation. Extracell Vesicles Circ Nucl Acids.

[43] Lötvall J, Hill AF, Hochberg F (2014). Minimal experimental requirements for definition of extracellular vesicles and their functions: a position statement from the International Society for Extracellular Vesicles. J Extracell Vesicles.

[44] Gardiner C, Di Vizio D, Sahoo S (2016). Techniques used for the isolation and characterization of extracellular vesicles: results of a worldwide survey. J Extracell Vesicles.

[45] Raposo G, Stoorvogel W (2013). Extracellular vesicles: exosomes, microvesicles, and friends. J Cell Biol.

[46] Liu H, Geng Z, Su J (2022). Engineered mammalian and bacterial extracellular vesicles as promising nanocarriers for targeted therapy. Extracell Vesicles Circ Nucl Acids.

[47] Witwer KW, Théry C (2019). Extracellular vesicles or exosomes? On primacy, precision, and popularity influencing a choice of nomenclature. J Extracell Vesicles.

[48] Smyth T, Petrova K, Payton NM (2014). Surface functionalization of exosomes using click chemistry. Bioconjug Chem.

[50] Ginini L, Billan S, Fridman E, Gil Z (2022). Insight into extracellular vesicle-cell communication: from cell recognition to intracellular fate. Cells.

[51] Ovčar A, Kovačič B (2024). Biogenesis of extracellular vesicles (EVs) and the potential use of embryo-derived evs in medically assisted reproduction. Int J Mol Sci.

[52] Gurunathan S, Kang MH, Kim JH (2021). A comprehensive review on factors influences biogenesis, functions, therapeutic and clinical implications of exosomes. Int J Nanomedicine.

[53] Panch T, Szolovits P, Atun R (2018). Artificial intelligence, machine learning and health systems. J Glob Health.

[54] Butler KT, Davies DW, Cartwright H, Isayev O, Walsh A (2018). Machine learning for molecular and materials science. Nature.

[55] Jordan MI, Mitchell TM (2015). Machine learning: trends, perspectives, and prospects. Science.

[56] Kwon SH, Dong L (2022). Flexible sensors and machine learning for heart monitoring. Nano Energy.

[57] Jovel J, Greiner R (2021). An introduction to machine learning approaches for biomedical research. Front Med.

[58] Walker A, Surda P (2019). Unsupervised learning techniques for the investigation of chronic rhinosinusitis. Ann Otol Rhinol Laryngol.

[59] Plemel JR, Stratton JA, Michaels NJ (2020). Microglia response following acute demyelination is heterogeneous and limits infiltrating macrophage dispersion. Sci Adv.

[60] Kler P (2021). The inversion factor, by linda bernardi, sanjay sarma and kenneth traub (MIT Press, 2018), 232 pages. Econ Rec.

[61] Mey A, Loog M (2023). Improved generalization in semi-supervised learning: a survey of theoretical results. IEEE Trans Pattern Anal Mach Intell.

[62] Chato L, Regentova E (2023). Survey of transfer learning approaches in the machine learning of digital health sensing data. J Pers Med.

[63] Thakur A, Santos Bezerra PC, Abhishek (2025). Quantum machine learning-based electrokinetic mining for the identification of nanoparticles and exosomes with minimal training data. Bioact Mater.

[64] Yu Y, Shao M, Jiang L (2021). Quantitative analysis of multiple components based on support vector machine (SVM). Optik.

[65] Cortes C, Vapnik V (1995). Support-vector networks. Mach Learn.

[66] Xu J, Ramos S, Vázquez D, López AM (2016). Hierarchical adaptive structural SVM for domain adaptation. Int J Comput Vis.

[67] Salazar-Rojas T, Cejudo-Ruiz FR, Calvo-Brenes G (2022). Comparison between machine linear regression (MLR) and support vector machine (SVM) as model generators for heavy metal assessment captured in biomonitors and road dust. Environ Pollut.

[68] Rivera-lopez R, Canul-reich J, Mezura-montes E, Cruz-chávez MA (2022). Induction of decision trees as classification models through metaheuristics. Swarm Evol Comput.

[69] Weinberg AI, Last M (2019). Selecting a representative decision tree from an ensemble of decision-tree models for fast big data classification. J Big Data.

[70] Rodríguez JJ, Kuncheva LI, Alonso CJ (2006). Rotation forest: a new classifier ensemble method. IEEE Trans Pattern Anal Mach Intell.

[71] Sorokin A, Zhu X, Lee EH, Cheng B (2023). SigOpt Mulch: an intelligent system for AutoML of gradient boosted trees. Knowl Based Syst.

[72] Beniaguev D, Segev I, London M (2021). Single cortical neurons as deep artificial neural networks. Neuron.

[73] Moldoveanu M (2023). *Explananda* and *explanantia* in deep neural network models of neurological network functions. Behav Brain Sci.

[74] Zanaty E (2012). Support vector machines (SVMs) versus multilayer perception (MLP) in data classification. Egypt Inform J.

[75] Lee J, Kim I (2022). Long-term stagnation monitoring using machine learning: comparison of artificial neural network model and convolution neural network model. Water Resour Manage.

[76] Ayub M, Kaka SI (2024). Enhanced first-break picking using hybrid convolutional neural network and recurrent neural networks. IEEE Trans Geosci Remote Sensing.

[77] LeCun Y, Bengio Y, Hinton G (2015). Deep learning. Nature.

[78] Wang Z, Zhou X, Kong Q (2024). Extracellular vesicle preparation and analysis: a state-of-the-art review. Adv Sci.

[79] Xiao X, Wu K, Yan A, Wang JG, Zhang Z, Li D (2021). Intelligent probabilistic system for digital tracing cellular origin of individual clinical extracellular vesicles. Anal Chem.

[80] Wang YT, Cai MD, Sun LL, Hua RN (2021). A rapid and facile separation-detection integrated strategy for exosome profiling based on boronic acid-directed coupling immunoaffinity. Anal Chem.

[81] (2023). Del Real Mata C, Jeanne O, Jalali M, Lu Y, Mahshid S. Nanostructured-based optical readouts interfaced with machine learning for identification of extracellular vesicles. Adv Healthc Mater.

[82] Jalali M, Del Real Mata C, Montermini L (2023). MoS_2_-Plasmonic nanocavities for raman spectra of single extracellular vesicles reveal molecular progression in glioblastoma. ACS Nano.

[83] Jin Y, Du N, Huang Y (2022). Fluorescence analysis of circulating exosomes for breast cancer diagnosis using a sensor array and deep learning. ACS Sens.

[84] Shen WX, Liu Y, Chen Y (2022). AggMapNet: enhanced and explainable low-sample omics deep learning with feature-aggregated multi-channel networks. Nucleic Acids Res.

[85] Pandian BJ, Noel MM (2018). Control of a bioreactor using a new partially supervised reinforcement learning algorithm. J Process Control.

[86] Ma X, Xiong H, Guo J (2023). Label-free breast cancer detection and classification by convolutional neural network-based on exosomes surface-enhanced raman scattering. J Innov Opt Health Sci.

[87] Greenberg ZF, Graim KS, He M (2023). Towards artificial intelligence-enabled extracellular vesicle precision drug delivery. Adv Drug Deliv Rev.

[88] Basile AO, Yahi A, Tatonetti NP (2019). Artificial intelligence for drug toxicity and safety. Trends Pharmacol Sci.

[89] Obrezanova O (2023). Artificial intelligence for compound pharmacokinetics prediction. Curr Opin Struct Biol.

[90] Cohn W, Melnik M, Huang C (2021). Multi-omics analysis of microglial extracellular vesicles from human Alzheimer’s disease brain tissue reveals disease-associated signatures. Front Pharmacol.

[91] Luo HT, Zheng YY, Tang J (2021). Dissecting the multi-omics atlas of the exosomes released by human lung adenocarcinoma stem-like cells. NPJ Genom Med.

[92] Lam SM, Zhang C, Wang Z (2021). A multi-omics investigation of the composition and function of extracellular vesicles along the temporal trajectory of COVID-19. Nat Metab.

[94] Yin H, Xie J, Xing S (2024). Machine learning-based analysis identifies and validates serum exosomal proteomic signatures for the diagnosis of colorectal cancer. Cell Rep Med.

[95] Reel PS, Reel S, Pearson E, Trucco E, Jefferson E (2021). Using machine learning approaches for multi-omics data analysis: a review. Biotechnol Adv.

[96] Asao T, Tobias GC, Lucotti S, Jones DR, Matei I, Lyden D (2023). Extracellular vesicles and particles as mediators of long-range communication in cancer: connecting biological function to clinical applications. Extracell Vesicles Circ Nucl Acids.

[97] Eitan E, Thornton-Wells T, Elgart K (2023). Synaptic proteins in neuron-derived extracellular vesicles as biomarkers for Alzheimer’s disease: novel methodology and clinical proof of concept. Extracell Vesicles Circ Nucl Acids.

[98] Zhou S, Hu T, Han G (2020). Accurate cancer diagnosis and stage monitoring enabled by comprehensive profiling of different types of exosomal biomarkers: surface proteins and miRNAs. Small.

[99] Wen Y, Zhang XW, Li YY, Chen S, Yu YL, Wang JH (2022). Ultramultiplex NaLnF_4 _nanosatellites combined with ICP-MS for exosomal multi-miRNA analysis and cancer classification. Anal Chem.

[100] Shin H, Oh S, Hong S (2020). Early-stage lung cancer diagnosis by deep learning-based spectroscopic analysis of circulating exosomes. ACS Nano.

[101] Yang Z, Tian T, Kong J, Chen H (2025). ChatExosome: an artificial intelligence (AI) agent based on deep learning of exosomes spectroscopy for hepatocellular carcinoma (HCC) diagnosis. Anal Chem.

[102] Sanchez-Manas JM, Perez de Gracia N, Perales S, Martinez-Galan J, Torres C, Real PJ (2024). Potential clinical applications of extracellular vesicles in pancreatic cancer: exploring untapped opportunities from biomarkers to novel therapeutic approaches. Extracell Vesicles Circ Nucl Acids.

[103] Zheng J, Zhou R, Wang B (2024). Electrochemical detection of extracellular vesicles for early diagnosis: a focus on disease biomarker analysis. Extracell Vesicles Circ Nucl Acids.

[104] Laine RF, Arganda-Carreras I, Henriques R, Jacquemet G (2021). Avoiding a replication crisis in deep-learning-based bioimage analysis. Nat Methods.

[105] Heil BJ, Hoffman MM, Markowetz F, Lee SI, Greene CS, Hicks SC (2021). Reproducibility standards for machine learning in the life sciences. Nat Methods.

[106] Karimzadeh M, Hoffman MM (2018). Top considerations for creating bioinformatics software documentation. Brief Bioinform.

[107] Challen R, Denny J, Pitt M, Gompels L, Edwards T, Tsaneva-Atanasova K (2019). Artificial intelligence, bias and clinical safety. BMJ Qual Saf.

[108] Char DS, Abràmoff MD, Feudtner C (2020). Identifying ethical considerations for machine learning healthcare applications. Am J Bioeth.

[109] Morley J, Machado CCV, Burr C (2020). The ethics of AI in health care: a mapping review. Soc Sci Med.

[110] Boateng D, Chu K, Smith ZJ, Du J, Dai Y (2024). Deep learning-based size prediction for optical trapped nanoparticles and extracellular vesicles from limited bandwidth camera detection. Biomed Opt Express.

[111] Dong Z, Liu X, Zhou S (2024). Microsphere lens array embedded microfluidic chip for SERS detection with simultaneous enhancement of sensitivity and stability. Biosens Bioelectron.

[112] Lin W, Yuan L, Gao Z (2023). An integrated sample-to-answer SERS platform for multiplex phenotyping of extracellular vesicles. Sens Actuators B Chem.

[113] Ma CY, Zhai Y, Li CT (2024). Translating mesenchymal stem cell and their exosome research into GMP compliant advanced therapy products: promises, problems and prospects. Med Res Rev.

